# Pharmacological and Genotoxic Properties of Polyphenolic Extracts of *Cedrela odorata* L. and *Juglans regia* L. Barks in Rodents

**DOI:** 10.1155/2015/187346

**Published:** 2015-04-05

**Authors:** Dulce Carolina Almonte-Flores, Norma Paniagua-Castro, Gerardo Escalona-Cardoso, Martha Rosales-Castro

**Affiliations:** ^1^CIIDIR-IPN Unidad Durango, Sigma 119 Fraccionamiento 20 de Noviembre, 34220 Durango, DGO, Mexico; ^2^Escuela Nacional de Ciencias Biológicas, Instituto Politécnico Nacional, Wilfrido Massieu s/n, Esquina Manuel L. Stampa, Colonia Unidad Profesional Adolfo López Mateos, Delegación Gustavo A. Madero, 07738 Ciudad de México, DF, Mexico

## Abstract

Evaluation of the phenolic compounds and antioxidant activity of *Cedrela odorata* L. and *Juglans regia* L. bark extracts was performed *in vitro*. *Juglans regia* showed greater extract concentration and higher antioxidant activity. Hypoglycemic activity in rats was assessed by generating a glucose tolerance curve and determining the area under the curve (AUC). Diabetes was later induced by an injection with streptozotocin (65 mg/kg of b.w.) and confirmed after 24 hours. The extract was administered (200 mg/kg b.w.) over 10 days, and blood glucose was monitored and compared with a control group. The glucose AUC showed a hypoglycemic effect of *J. regia* and *C. odorata* in normal rats. Both extracts reduced hepatic lipid peroxidation in diabetic rats. Polyphenolic extracts reduced cholesterol levels in a hypercholesterolemic mouse model and decreased hepatic lipid peroxidation. Polyphenolic extract doses of 100 and 200 mg/kg b.w. were administered alone or with cyclophosphamide (CPA) 50 mg/kg ip, which was used as a positive control. Analyses were performed using leukocytes in a comet assay after 4 and 24 h of treatment. Genotoxic effects were evaluated by the comet assay, which showed that while *J. regia* extract had no effect, *C. odorata* extract induced slight damage at 200 mg/kg, with the formation of type 0 and 1 comets.

## 1. Introduction

*Juglans regia* L. (Juglandaceae) comprises several species and is widely distributed throughout the world. The walnut tree is a well-known deciduous species that is found primarily in temperate areas and is cultivated commercially throughout southern Europe, northern Africa, eastern Asia, the United States, and western South America. Furthermore, the dry seeds (nuts) are not the only component used; green walnuts, shells, bark, green husks (pericarps), and leaves have all been used in the cosmetic and pharmaceutical industries [1]. The steam bark is used as an astringent, digestive, diuretic, and tooth cleaner [2]. Leaves are used in the treatment of hypertension and diabetes in southeastern Morocco (Errachidia province) [3].

*Cedrela odorata* L. (Meliaceae) is a valuable timber tree that is native to the tropical region of America. The tree is known as red cedar, “cedro rojo,” and Spanish cedar. In traditional medicine, a bark infusion is used as a remedy for diarrhea, fever, anti-inflammation, vomiting, hemorrhage, and indigestion [4]. In Africa, the decoction of the bark is used against malaria and/or fever [5].

Phenolic compounds are secondary plant metabolites which are important determinants in the sensory and nutritional quality of fruits, vegetables, and other plants [6, 7]. Several studies have shown that phenolic compounds are the major bioactive phytochemicals that confer human health benefits. In fact, a direct relationship between antioxidant activity and the total phenolic content of numerous seeds, fruits, and vegetables has been reported [1]. Many varieties of medicinal plants are recognized as a source of natural antioxidants that can protect from oxidative stress and thus play an important role in the chemoprevention of diseases [7]. Plants can be used in the food industry for their organoleptic and nutritional qualities, as sources of antioxidants to preserve food quality, and also for medicinal purposes as they remain a major source of healthcare and disease prevention for a great part of the world's population [8].

Some substances produced by plants have been studied and characterized. There are reports that extracts of* J. regia* and* C. odorata* have phenolic compounds in their composition, but insufficient toxicological and genotoxicological studies have been performed on these compounds. However, there has been a recent growing interest in the possible toxic, genotoxic, and mutagenic effects of phytochemicals that are used therapeutically [9]. The genotoxicity of plants can be measured with a group of techniques that include the micronucleus (MN), Ames test, AO/EB tint double, and the comet assay [10]. The comet assay, also referred to as the single cell gel electrophoresis (SCG or SCGE) assay, is a rapid, visual, and quantitative technique for measuring DNA damage in eukaryotic cells [11]. Under alkaline (pH > 13) conditions, the assay can detect single and double-stranded breaks, incomplete repair sites, alkali labile sites, and also possibly both DNA-protein and DNA-DNA cross-links in virtually any eukaryotic cell population that can be obtained as a single cell suspension [11–13].

Genetic toxicology tests are assays designed to detect compounds that induce genetic damage directly or indirectly. Genetic damage plays an important role in many malignancies and may also induce heritable effects that can lead to birth defects. Thus, identifying the genotoxic effects of any agent is important for the risk/benefit assessment of its potential use in humans [13].

Despite the studies on phenolic compounds in* Juglans regia*, there is little information about the hypoglycemic and hypocholesterolemic properties of their barks and the relationship between the pharmacological effects of the phenolic compounds and their genotoxic effects. A recent study on* C. odorata* reports hypoglycemic activity from a hydro alcoholic extract without toxic effects. Nevertheless, phenolic compounds were not tested, thus underlining the importance of the present research [14].

## 2. Materials and Methods

### 2.1. Chemicals

Ethanol, methanol, chloroform, and ethyl acetate were purchased from Merck KGaA (Darmstadt, Germany). Catechin, gallic acid, linoleic acid, 2,2-diphenyl-1-picrylhydrazyl (DPPH), Folin-Ciocalteu reagent, vanillin, linoleic acid, *β*-carotene, streptozotocin, and tyloxapol (Triton WR-1339) were acquired from Sigma Chemical Co. All other reagents were of analytical grade. Kits for the determination of total cholesterol, cholesterol HDL, and triglycerides were from RANDOX.

### 2.2. Plant Material

The bark of* C. odorata* was collected at Tezonapa, Veracruz, and* J. regia* bark was collected at Cuencamé, Durango, México. Botanical specimens were deposited at the Herbarium CIIDIR-IPN, Durango, with voucher numbers 43329 and 43328, respectively. The samples were dried at room temperature (24°C) and milled (mesh 60).

### 2.3. Extract Preparation

The barks were soaked independently in 96% ethanol and 70% ethanol (ethanol/water 70 : 30 v/v) for 48 h in the dark, followed by filtration through Whatman no. 1 filter paper. In a third extract, 100 mL of hot water was added to 10 g of bark and subjected to boiling for 15 min. The crude extracts were concentrated in a rotary evaporator under reduced pressure to finally obtain the crude extracts. A portion of the concentrated ethanolic extract (70 : 30) was subjected to liquid partition with ethyl acetate (3 × 50 mL). The organic phase was evaporated to dryness under vacuum at 35°C and identified as the fourth extract.

### 2.4. Evaluation of the Phenolic Compounds

The concentration of the total phenolic (TP) was determined by the Folin-Ciocalteu colorimetric method [15]. Measurements were carried out in duplicate and calculations based on a calibration curve were obtained with gallic acid. The total phenolic concentration was expressed as milligrams of gallic acid equivalents (GAE) per gram of dry extract. The concentrations of total flavonoids (TF) and proanthocyanidins (PAC) were determined according to a previous study [16]. Results were expressed as milligrams of catechin equivalents (CE) per gram of dry extract.

### 2.5. Determination of Antioxidant Activity

The DPPH assay was determined according to a previous study [17] with some modifications. For each extract, three concentrations were tested: 100, 200, and 300 *μ*g/mL. Samples dissolved in methanol (50 *μ*L) were mixed with 1950 *μ*L of a methanolic solution of DPPH (2.4 mg/100 mL). After incubation for 30 min at room temperature (±25°C), the decrease in absorbance was measured at 515 nm in a Varian 50 Bio Spectrophotometer. The antioxidant (antiradical) activity was expressed as the median effective concentration EC_50_ in *μ*g/mL.

The *β*-carotene bleaching method was performed according to a previous study [18] based on the ability of the different extracts to decrease the oxidative bleaching of *β*-carotene in a *β*-carotene/linoleic acid emulsion [19]. Briefly, 0.6 mg of *β*-carotene was dissolved in 5 mL of chloroform and this solution was added to a beaker containing 20 mg of linoleic acid and 100 mg of Tween 40. After the removal of chloroform by evaporation, 50 mL of distilled water was added to the flask under vigorous stirring (emulsion A). Subsequently, emulsion B was elaborated with 20 mg of linoleic acid, 100 mg of Tween 40, and 50 mL of water. In a test tube, 0.2 mL of ethanol was added to 5 mL of emulsion B for use as a blank. Afterwards, a sample was prepared with 0.2 mL of extract dissolved in ethanol and 5 mL of emulsion A. The sample was stirred, and the absorbance was immediately read at 470 nm. The sample was then heated at 50°C for 120 min and allowed to cool in water until it reached 20°C before the final absorbance was read.

The antioxidant activity (AA) was calculated according to the following expression:

(1) $$\begin{array}{c} \%\text{AA}=\left[\frac{A_{S\left[120\right]}-A_{c\left[120\right]}}{A_{c\left[0\right]}-A_{c\left[120\right]}}\right]\times 100, \end{array}$$

where *A*_*S*(120)_ is the sample absorbance at 120 min, *A*_*c*(120)_ is the control absorbance at 120 min, and *A*_*c*(0)_ is the control absorbance at time zero. The results were expressed as % inhibition.

### 2.6. Animals

Healthy Wistar rats were obtained from the Biorepository of the National School of Biological Sciences (National Polytechnic Institute) and ICR mice were obtained from the National Institute of Hygiene. All experiments were approved by the Laboratory Animal Care Committee of the National School of Biological Sciences (National Polytechnic Institute) and were conducted in compliance with the Mexican Official Standard (NOM—062-200-1999) technical specifications for the production, care, and use of laboratory animals. The animals were group-housed in polycarbonate cages in a controlled environment at a constant temperature (21 ± 2°C) with 12 h light/dark cycles and access to food and fresh water* ad libitum*.

### 2.7. Evaluation of Hypoglycemic Properties in Rats

The hypoglycemic activity was evaluated using a glucose tolerance test in female Wistar rats weighing 300 g. The study animals were fasted for 12 h before testing and basal blood samples were obtained from the tip of the tail. Animals received 1 mL of extract by gavage (doses 200 mg/kg body weight) at time zero. After fifteen minutes, all animals received an oral dose of 35% glucose (3 g/kg body weight). The control group was administered only glucose. At 0, 30, 60, 90, and 120 min after glucose administration, blood glucose was measured using a glucometer (Accu-Check Performa with Softclix, Roche) and then the area under the curve was calculated to estimate the glucose tolerance.

### 2.8. Hypoglycemic Effect

The hypoglycemic effect of the extracts was tested in female Wistar rats (5 animals/dose of extract) with previously induced type I diabetes by intraperitoneal streptozotocin injection (STZ, 65 mg/kg of body weight, in citrate buffer, pH 4.4) in which the hyperglycemia was confirmed by measuring the glucose levels in the blood 24 hours after injection. Five days later, animals received 200 mg/kg* C. odorata* or* J. regia* ethyl acetate extracts for 10 days. Blood glucose was determined (Accu-Check Performa with Softclix, Roche) and compared with a control group. After the animals were euthanized, fasted liver samples were taken for lipid peroxidation assays.

### 2.9. Hypocholesterolemic Activity in Mice

The hypocholesterolemic activity was tested in mice using the tyloxapol model [20]. Male mice were initially administered 100 mg/kg of either* C. odorata* or* J. regia *extract, and after 2 hours animals received 40 mg/kg of tyloxapol i.p. On the next day, a second dose of tyloxapol was administered to mice, and after two hours, blood samples were collected by retroorbital puncture to determine the lipid profile using specific enzymatic-colorimetric kits (Randox, Crumlin, UK). The samples were centrifuged at 3500 rpm for 15 min at 4°C. Plasma levels of total cholesterol (TC), triglycerides (TG), and HDL-cholesterol (HDL) were determined. Animals were euthanized and liver samples were quickly taken for lipid peroxidation assay.

### 2.10. Hepatic Lipid Peroxidation Assay

Oxidative stress levels were evaluated by the concentration of malondialdehyde (MDA) in the liver according to a previous study [21]. Briefly, 50 mg of liver was homogenized with 0.5 mL cold phosphate buffer (pH 7.0) and 0.5 mL of this solution was added to 1 mL of TBARS reagent which contained trichloroacetic acid (TCA), thiobarbituric acid (TBA), and HCL (15% (w/v), 0.375% (w/v), and 0.25 N, resp.). Samples were then placed in a water bath at boiling temperature for 1 h, cooled, and centrifuged at 3000 rpm for 15 min, and then the supernatant was removed and the absorbance read at 532 nm. The blank did not contain sample. The MDA concentration was calculated using the extinction coefficient of 1.56 × 10^−5 ^M^−1 ^cm^−1^. The results were expressed in mmol MDA/g tissue.

### 2.11. Genotoxic Assay

Thirty-six male mice weighing 25–30 g were divided into six experimental groups of six animals each. The extracts were suspended in 1% Tween-80 aqueous solution and 0.3 mL was administered by gavage at doses of 100 and 200 mg/kg body weight. The doses were chosen on the basis of their pharmacological activity. The negative group received 1% Tween-80 aqueous solution, and the positive control group received an intraperitoneal injection of cyclophosphamide (CPA) at 50 mg/kg body weight.

### 2.12. Comet Assay

The comet assay was carried out as previously described [13, 22] with some modifications. A peripheral blood sample obtained by retroorbital puncture was collected at 4 and 24 h after treatment. An aliquot of 30 *μ*L of heparinized blood was mixed with 140 *μ*L of 0.5% low-melting-point agarose at 37°C and rapidly spread onto two microscope slides per animal that were precoated with a layer of 1.5% of normal-melting-point agarose. After coverslip removal, the slides were placed in cold lysing solution (2.5 M NaCl, 2.5 M EDTA, 10 mM Tris, sodium succinate, 1% Triton X100, and 10% dimethyl sulfoxide, pH 10.5 adjusted with NaOH) at 4°C for 3 h and finally with buffer (0.4 M NaOH and 200 mM EDTA) at 4°C for 20 min. The slides were then subjected to electrophoresis which was run for 20 min at 300 mA and 25 V. Afterwards, the slides were washed and neutralized with buffer solution (0.4 M Tris-HCl, pH 7.5), dried at room temperature, fixed in ethanol-neutralization buffer (50 : 50) for 10 min, and dried and stored before staining with ethidium bromide. The extent and distribution of DNA damage were evaluated by examining at least 100 randomly selected cells (50 cells per coded slide) per animal in a blind analysis (six mice per group). These cells were scored visually according to tail size and grouped into the following four classes: class 0, no tail; class 1, tail shorter than the diameter of the head (nucleus); class 2, tail length 1-2 times the diameter of the head; and class 3, tail length more than twice the diameter of the head [13]. The total comet score was calculated by the following equation modified from Matić et al. [9]: (% of cells in class 0 × 0) + (% of cells in class 1 × 1) + (% of cells in class 2 × 2) + (% of cells in class 3 × 3).

### 2.13. Statistical Analysis

The results are expressed as the mean and the standard deviation. One-way and two-way analyses of variance (ANOVA) were performed followed by multiple comparisons of the means with the Fisher-LSD test at a significance level of *α* = 0.5. The statistical analysis software package (Statistica 7) was used for these analyses.

## 3. Results

In* C. odorata*, the maximum extraction yield was obtained with 70% ethanol solvent (5.89%), while in* J. regia* the water extraction gave the highest yield (21.4%). The range of TP was from 203.2 to 577.3 mg GAE/g extract, TF from 94.3 to 374.8 mg CE/g extract, and PAC from 144.6 to 548.7 mg CE/g extract. In* J. regia*, TF and PAC content was higher than in* C. odorata*. A higher antioxidant capacity was obtained with the 70% ethanol extract in* C. odorata*, while ethyl acetate extracts of* J. regia* were shown to have the highest antioxidant capacity for this plant. Significant differences (*P* < 0.05) were observed between plants and extraction solvents in terms of yield, the concentrations of TP, TF, and PAC, and antioxidant capacity (Table 1).

**Figure 1:**
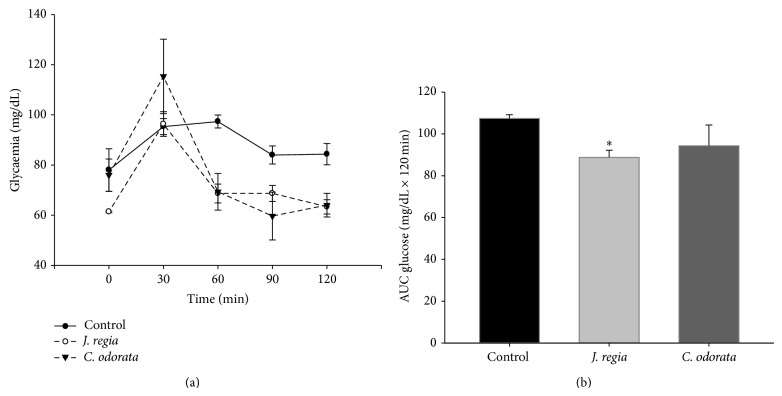
Glucose tolerance test in rats. (a) Glucose levels. (b) Area under the glucose curve. Two-way RM ANOVA for (a) and one-way ANOVA for (b).  ^∗^ *P* < 0.05, significant difference compared with the control group.

In regard to the glucose tolerance test in rats,* C. odorata* and* J. regia *extracts significantly decreased blood glucose levels (94.3 ± 17.2 g/L min and 88.8 ± 5.8 g/L min, resp.) (Figure 1) compared with the control group. Extracts of* J. regia* showed the highest hypoglycemic activity (*P* < 0.05).

In the present study, STZ-induced diabetic rats showed elevated blood glucose levels compared with control rats (Table 2). The administration of* J. regia* and* C. odorata* extracts reduced blood glucose levels compared with the diabetic control group. The* J. regia* extract was more effective in reducing blood glucose than the* C. odorata* extract (*P* < 0.05).

In the hypercholesterolemic mice model, the group administered tyloxapol (Tx group) showed elevated TC, TG, and HDL-cholesterol compared with the control group (*P* < 0.05). The groups treated with* C. odorata* and* J. regia* extracts showed significantly reduced CT (8.6 ± 3.7 and 11 ± 6.5 mmol/L, resp.) with respect to the Tx group (29.03 ± 3.42 mmol/L); no differences were observed with the control group (5.1 ± 3.7 mmol/L) with regard to the levels of TG and HDL-cholesterol with either extract.

**Figure 2:**
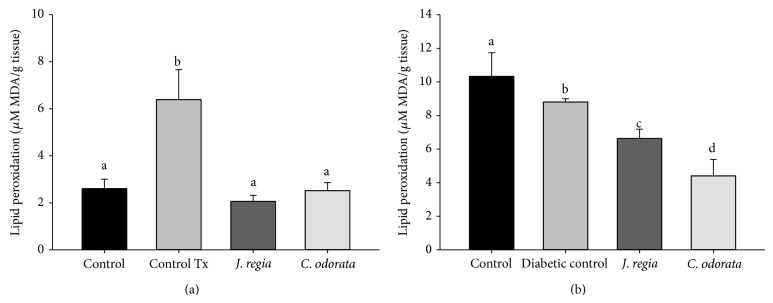
Hepatic lipid peroxidation assay. (a) Hypercholesterolemic mice treated with* C. odorata* or* J. regia* extracts. (b) Diabetic rats treated with* C. odorata* or* J. regia* extracts during 10 days. Different letters between groups on each figure indicate a significant difference (*P* < 0.05), one-way ANOVA; Tx: tyloxapol.

For the hepatic lipid peroxidation assay, the diabetes induction with STZ in rats and the administration of tyloxapol in mice both resulted in a significant (*P* < 0.05) increase in the levels of MDA compared with their respective controls. The administration of* C. odorata* and* J. regia* extracts reduced MDA levels in liver samples (*P* < 0.05) with respect to the streptozotocin and tyloxapol groups (Figure 2).

**Figure 3:**
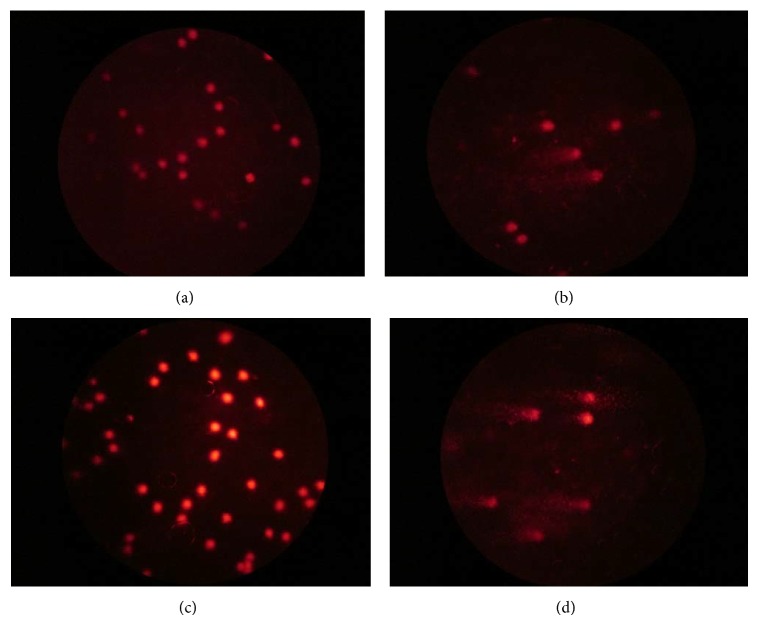
Comet assay images in leucocytes of mice (400x). (a) Negative control group, (b) CPA group, (c) extract of* J. regia* at 200 mg/kg, and (d) extract of* C. odorata* at 200 mg/kg.

The results of the genotoxicity assay are shown in Table 3 and Figure 3. DNA damage was tested with the comet assay with extracts of* J. regia* and* C. odorata.* In this study, it was demonstrated that* in vivo* exposure to the* J. regia* extract itself did not induce DNA damage at doses of 100 and 200 mg/kg in leucocytes of mice. A significant difference (*P* > 0.05) was not present in the negative control group, whereas in the CPA group a significant difference was observed (*P* < 0.05).

The negative control group showed high levels of genotoxicity according to the comet classes detected, with a predominance of damage classes 2 and 3 (maximum damage) which was significantly different (*P* < 0.05) to the CPA-treated group (Figure 3).

The* C. odorata* extract at a dose of 100 mg/kg did not show a significant difference with the negative control. However, at 200 mg/kg, light damage was observed with the formation of comets of type 1 with a total number of damaged cells of 109.50 ± 30.48 and 29.33 ± 15.88 at 4 and 24 hours, respectively; this dose also showed differences with the negative control (*P* < 0.05). The observed damage level was not the same in the CPA group where the total number of damaged cells was 170.16 ± 12.31 and 96.83 ± 8.93 at 4 and 24 hours, respectively.

On the other hand, a separate analysis between the blood samples collected at 4 and 24 hours showed that there were indeed significant differences (*P* < 0.05).

## 4. Discussion

The health benefits of* J. regia* are attributed principally to chemical composition. Previous works reported that extracts from leaves, stems, pericarps, fruits, and barks have phenolic compounds with biologic importance [23].

This work showed that* J. regia* extracts have a high concentration of phenolic compounds (phenolics, flavonoids, and proanthocyanidins) and also have antioxidant activity. These biological properties have been studied by other authors [24] who reported antiglycation properties and the antioxidant potential of* J. regia*. It was also mentioned that in diabetes the advanced glycation end products (AGEs) are formed at an accelerated rate due to hyperglycemia, thus resulting in the promotion and progression of diabetic complications such as nephropathy, cardiovascular disease, and atherosclerosis. In the same study, an* in vitro* glycation assay demonstrated that the crude methanolic extracts of* J. regia* could inhibit glycation and oxidation reactions [24]. For this reason, the bark of* J. regia* could have therapeutic potential against diabetic complications. Our results showed that* J. regia* extracts significantly decreased (*P* < 0.05) blood glucose levels at the beginning of the treatment in diabetic rats. It is noteworthy that by the end of treatment in the diabetic group two animals died due to physical deterioration due to diabetes, whereas animals treated with the extracts did not die. A significant difference in the weights of the animals was observed because the diabetic animals had a greater weight loss than animals receiving extract. It was also reported that flavonoids have the ability to improve diabetic conditions by decreasing blood glucose levels [25]. The results showed that* J. regia* and* C. odorata* extracts have high levels of these compounds (374.8 ± 7.6 and 144.6 ± 10.5 mg CE/g extract, resp.).

In most cases, hypercholesterolemia and hypertriglyceridemia are the most common lipid abnormalities in diabetes and confer increased risk for the premature development of atherosclerosis and cardiovascular diseases; therefore, we focused on these alterations. Our results showed that* C. odorata* and* J. regia* extracts significantly decreased (*P* < 0.05) cholesterol levels in hypercholesterolemic mice compared with the control group. Hyperglycemia is a well-known cause for elevated free radical levels, followed by the production of reactive oxygen species (ROS) which can lead to increased lipid peroxidation, alter antioxidant defenses, and further impair glucose metabolism in biological systems [26]. This can cause damage particularly to the liver, kidney, and pancreas. The level of lipid peroxidation (TBARS) and reactive oxygen species are common markers of oxidative stress. Malondialdehyde (MDA) is one of the end products of lipid peroxidation [27]. Our results showed that* C. odorata* and* J. regia* extracts significantly decreased MDA levels in diabetic and hypercholesterolemic animals, probably due to the bioactive compounds with antioxidant activity present in the extracts.

The toxicity results showed that* J. regia* extract was not considered a genotoxic agent at the doses tested in this study. On the other hand,* C. odorata* extract did not show a genotoxic effect at 100 mg/kg, but a low level of genotoxicity was observed at 200 mg/kg. Some studies that have analyzed other plant extracts have also demonstrated an inverse dose-dependent relationship. As such, the lack of a dose-response relationship might be attributed to the activation of different cellular pathways depending on the tested dose. The use of mutagenic drugs for cancer chemotherapy, such as cyclophosphamide (CPA), is a routine practice. Therefore, identifying chemopreventive agents is important for the risk/benefit assessment of their potential use in humans [13]. The search for medical treatments based on alternative medicine has increased significantly and further knowledge about the plants commonly used in folk medicine is extremely important. Antimutagenic activities have been correlated with the presence of certain phytochemical substances such as flavonoid compounds. A relationship has been reported between structure and activity, both for mutagenic activity and for protection of the genetic material. Most of these health-beneficial effects are thought to derive from effective antioxidant and free-radical-scavenging properties, as well as the ability to regulate many cellular enzymatic functions [9].

In conclusion, the present work demonstrated that extracts of* C. odorata* and* J. regia* bark have high concentrations of phenolic compounds with antioxidant activity and also showed hypoglycemic and hypocholesterolemic effects in animals. Furthermore, we found novel information about the genotoxic effects of* J. regia* and* C. odorata* extracts.

## Figures and Tables

**Table 1:** Concentration of phenolic compounds and antioxidant capacity in *C*. *odorata* and *J*. *regia* bark extracts.

Solvent	Yield %	TP mg GAE/g extract	TF mg CE/g extract	PAC mg CE/g extract	DPPH (EC_50_) *µ*g/mL	*β*-Carotene (% AA)
*Cedrela odorata *						
Water	5.07	203.2 ± 18.0^a^	94.3 ± 10.3^a^	144.6 ± 16.1^a^	1385.4 ± 31.8^a^	7.1 ± 0.5^a^
Ethanol 96%	2.28	332.4 ± 10.9^b^	144.3 ± 10.3^b^	180.0 ± 9.1^ab^	645.5 ± 22.0^b^	9.5 ± 0.5^b^
Ethanol 70%	5.89	549.6 ± 20.7^c^	144.6 ± 10.5^b^	266.7 ± 10.7^c^	654.2 ± 21.9^b^	10.4 ± 0.3^c^
Ethyl acetate	1.30	304.9 ± 12.1^d^	186.4 ± 19.7^c^	313.2 ± 11.3^c^	739.1 ± 54.4^c^	9.5 ± 0.5^b^
*Juglans regia *						
Water	21.40	405.7 ± 23.0^e^	257.4 ± 17.0^d^	198.7 ± 2.7^b^	517.7 ± 12.2^d^	14.0 ± 3.8^d^
Ethanol 96%	6.76	577.3 ± 24.9^c^	296.5 ± 10.0^e^	538.9 ± 72.6^d^	323.2 ± 4.0^e^	13.4 ± 1.4^d^
Ethanol 70%	10.54	570.3 ± 13.4^c^	321.2 ± 15.8^e^	548.7 ± 59.0^d^	373.8 ± 4.9^e^	12.5 ± 0.9^e^
Ethyl acetate	1.05	568.7 ± 15.3^c^	374.8 ± 7.6^f^	443.0 ± 24.0^e^	331.2 ± 4.7^e^	14.1 ± 1.0^d^

Each value represents the mean of *n* = 4  ± standard deviation. Different letters between groups in each column indicate a significant difference (*P* < 0.05) assessed using the Fisher-LSD test.

**Table 2:** Blood glucose levels during the study period (10 days) in Wistar rats.

Group	Blood glucose (mg/dL)			
	Day 1	Day 2	Day 3	Day 10
Control	90.6 ± 17.0^a^	95.4 ± 5.5^a^	89.0 ± 7.8^a^	73.0 ± 35.3^a^
Diabetic control	411.4 ± 35.2^bc^	434.4 ± 37.9^b^	439.0 ± 35.2^b^	456.6 ± 34.4^b^
*C*. *odorata *	431.8 ± 81.6^b^	442.4 ± 55.7^b^	415.8 ± 42.2^b^	447.6 ± 30.0^b^
*J*. *regia *	360.2 ± 36.0^c^	390.2 ± 57.0^b^	426.0 ± 22.7^b^	446.3 ± 3.05^b^

Different letters between groups on each day indicate a significant difference (*P* < 0.05), Fisher-LSD test.

**Table 3:** DNA damage according to the comet assay for the assessment of the genotoxicity of *J*. *regia* and *C*. *odorata. *

Treatments		Comet class			
	Total	0	1	2	3
*Blood samples* (4 h)					
Negative control	40.5 ± 21.41^a^	77.0 ± 9.38^a^	10.7 ± 4.08^a^	7.2 ± 5.60^a^	5.2 ± 3.54^a^
CPA (50 mg/kg)	170.2 ± 12.31^b^	5.0 ± 2.28^b^	39.0 ± 7.01^b^	36.8 ± 5.52^b^	19.2 ± 3.12^b^
*J. regia * (100 mg/kg)	64.0 ± 52.44^c^	60.8 ± 25.4^a^	20.8 ± 6.83^bc^	12.0 ± 11.22^c^	6.4 ± 8.29^a^
*C. odorata * (100 mg/kg)	31.8 ± 8.93^a^	73.3 ± 6.56^a^	19.8 ± 3.86^bc^	5.5 ± 5.24^a^	0.3 ± 0.18^c^
*J. regia * (200 mg/kg)	58.7 ± 20.83^a^	65.5 ± 8.07^a^	15.5 ± 5.00^a^	13.8 ± 9.97^c^	5.2 ± 3.60^a^
*C. odorata * (200 mg/kg)	109.5 ± 30.48^b^	39.2 ± 10.94^c^	25.7 ± 4.67^c^	21.7 ± 3.26^d^	13.5 ± 9.28^b^
*Blood samples* (24 h)					
Negative control	15.0 ± 3.79^a^	87.8 ± 3.37^a^	9.8 ± 3.54^a^	1.8 ± 0.75^a^	0.5 ± 0.83^a^
CPA (50 mg/kg)	96.8 ± 8.93^b^	29.5 ± 5.08^b^	47.3 ± 7.14^c^	20.0 ± 4.56^b^	3.2 ± 1.94^b^
*J. regia * (100 mg/kg)	9.4 ± 7.53^a^	90.8 ± 7.25^a^	8.6 ± 6.54^a^	0.4 ± 0.89^a^	0^a^
*C. odorata * (100 mg/kg)	12.3 ± 4.17^a^	88.8 ± 3.43^a^	10.0 ± 2.96^a^	1.2 ± 1.16^a^	0^a^
*J. regia * (200 mg/kg)	11.8 ± 4.40^a^	89.2 ± 3.86^a^	9.8 ± 3.48^a^	1.0 ± 0.89^a^	0^a^
*C. odorata * (200 mg/kg)	29.3 ± 15.88^c^	74.7 ± 11.96^c^	21.2 ± 8.95^b^	3.8 ± 0.86^c^	0.2 ± 0.40^a^

Mean ± standard error. Different letters between groups in each column correspond to significant differences at 4 and 24 hours. *P* < 0.5, one-way ANOVA; Fisher LSD, comparing the treatments separately at 4 and 24 hours.
